# Novel Heterozygous Deletion in Retinol Dehydrogenase 12 (*RDH12*) Causes Familial Autosomal Dominant Retinitis Pigmentosa

**DOI:** 10.3389/fgene.2020.00335

**Published:** 2020-04-08

**Authors:** Hajrah Sarkar, Adam M. Dubis, Susan Downes, Mariya Moosajee

**Affiliations:** ^1^Development, Ageing and Disease Theme, UCL Institute of Ophthalmology, London, United Kingdom; ^2^Department of Genetics, Moorfields Eye Hospital NHS Foundation Trust, London, United Kingdom; ^3^Nuffield Department of Clinical Neuroscience, University of Oxford, Oxford, United Kingdom; ^4^Department of Ophthalmology, Great Ormond Street Hospital for Children NHS Foundation Trust, London, United Kingdom

**Keywords:** RDH12, autosomal dominant, retinitis pigmentosa, adaptive optics, Leber congenital amaurosis

## Abstract

Mutations in the retinol dehydrogenase 12 (*RDH12*) gene are primarily associated with Leber congenital amaurosis (LCA) type 13, a severe early onset autosomal recessive retinal dystrophy. Only one family with a heterozygous variant, associated with mild retinitis pigmentosa (RP), has been reported. We report a novel heterozygous variant [(c.759del; p.(Phe254Leufs^∗^24)], resulting in a frameshift and premature termination identified in two unrelated individuals with familial autosomal dominant RP. Both heterozygous variants are associated with a late onset RP phenotype, suggesting a possible genotype-phenotype correlation.

## Introduction

Retinol dehydrogenase 12 (RDH12) is an NADPH-dependent retinal reductase that catalyzes the reduction of all-trans retinal to all-trans retinol, and is expressed in photoreceptor inner segments (Belyaeva et al., 2005). *RDH12* has 7 coding exons, is located on chromosome 14q24.1 and encodes a 316 amino acid protein. Mutations in *RDH12* are primarily associated with Leber congenital amaurosis (LCA), a severe early onset autosomal recessive retinal dystrophy, and accounts for 3.4–10.5% of all cases (Kumaran et al., 2017). Patients present with signs of early onset central visual loss, nystagmus, not reaching or difficulty finding dropped objects and nyctalopia. This is a progressive disorder with significant decline from 10 years of age, which leads to complete blindness in adulthood. *RDH12*-LCA is characterized by macular atrophy, which extends peripherally in a variegated pattern corresponding to the retinal vasculature, and midperipheral pigmentary retinopathy (Aleman et al., 2018; Fahim et al., 2019). According to the Human Gene Mutation Database (HGMD), 80 unique *RDH12* variants have been reported (HGMD public database accessed August 2019). One six-generation family with 19 affected members has been reported to harbor an autosomal dominant variant, c.776del; p.(Glu260Argfs^∗^18), a single base pair deletion resulting in a frameshift and premature termination. Affected individuals displayed a milder late onset (average age of diagnosis was 28.5 years) retinitis pigmentosa (RP) phenotype, with intraretinal bone spicule pigmentation and attenuation of retinal arterioles (Fingert et al., 2008). Herein this report, we describe two unrelated individuals with autosomal dominant RP associated with a novel point deletion in *RDH12* with a similar phenotype. The patient study protocol adhered to the tenets of the Declaration of Helsinki, and received approval from the NRES Ethics Committee (REC12/LO/0141). Written informed consent was obtained from the individuals for the publication of any potentially identifiable images or data included in this article.

## Case Presentation

**FIGURE 1:**
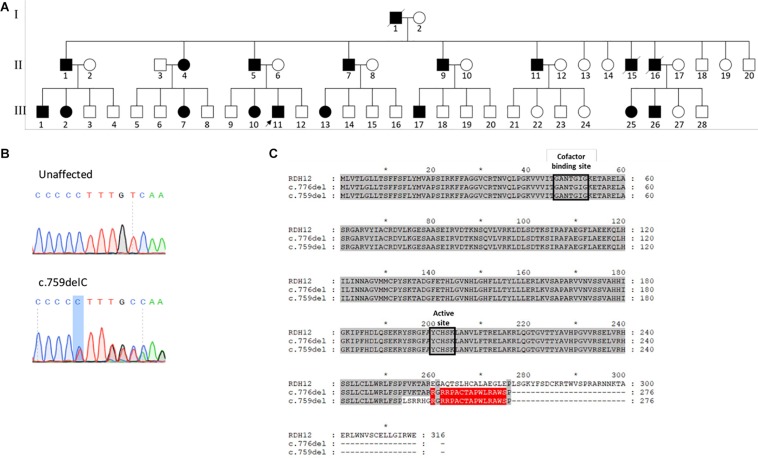
**(A)** Pedigree of family of patient 1 affected with autosomal dominant *RDH12*-retinitis pigmentosa (RP). Affected individuals are colored in black. Deceased individuals are indicated with a slash and patient 1 is indicated by an arrow. Children of unaffected parents did not have RP, and are not shown. **(B)** Sanger sequencing traces confirming heterozygous c.759del mutation. **(C)** Alignment of the RDH12 (UniProt Q96NR8) sequence with the previously reported c.776del variant and the current c.759del variant. Identical residues to the reference sequence are highlighted in gray, and identical residues between the two mutant sequences are highlighted in red.

**FIGURE 2:**
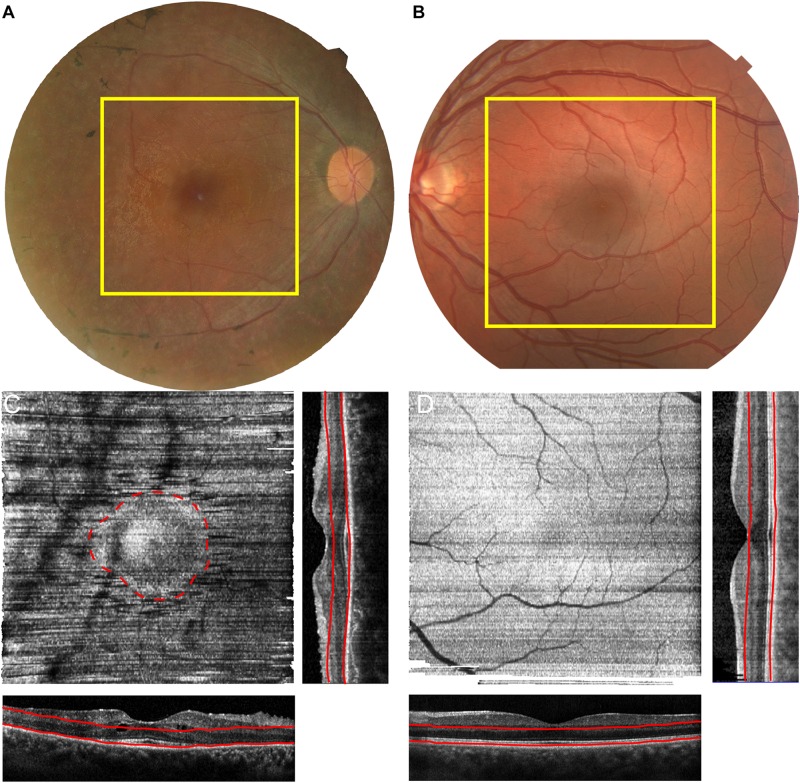
Identification of Residual Photoreceptors through *Enface* OCT analysis. Macular SD-OCT images were acquired for a 20° × 20° area in patient 1 **(A)** and age matched control **(B)**. The RPE layer was segmented, with manual correction and a maximum intensity slab was generated for both subjects **(C,D)**. The corresponding OCT, generated orthogonal OCTs are displayed on the side and bottom of each intensity projection. The red lines correspond to the location of the slab on the corresponding OCTs. The area of residual cones is highlighted in C (red dashed line).

Patient 1 is a 32-year-old Israeli man of mixed ethnicity, mother from Kurdistan and affected father from Tunisia, who presented with nyctalopia and field loss, but preserved central vision. He has an extensive positive family history exhibiting autosomal dominant inheritance with affected father, paternal grandfather and siblings (Figure 1A). His best corrected visual acuity with LogMAR was 0.04 in both eyes, fundus examination showed mild waxy disc pallor with retinal vessel attenuation and mid-peripheral bone-spicules. Dense spectral domain optical coherence tomography (SD-OCT) volumes were performed using the Heidelberg Spectralis (Heidelberg Engineering GmbH, Heidelberg, Germany). The area of SD-OCT is delineated by the yellow boxes on the fundus image in Figure 2A. The volume was acquired using high resolution, 1024 A-scan/B-scan and 193 B-scan over a 20 × 20° area. From this volume, the maximum intensity projection *enface* slab was generated (Chen et al., 2015) using the instrument software. Retinal layer segmentation was manually corrected for all B-scans by one observer (AMD). The slab was designed to measure the area of intact ellipsoid zone. This slab used the retinal pigment epithelium (RPE) contour and was defined as 10 μm above the RPE with a thickness of 100 μm (Figure 2C – red lines). The maximum intensity slab showed an area of hyper-reflectivity surrounded by a ring of lower reflectance (dashed red line – Figure 2C). This pattern is due to the increased reflectivity from the intact ellipsoid zone band of the structural OCT and identifies the area of intact photoreceptors. This circular pattern is more reminiscent of RP than classic recessive *RDH12* retinopathy (Aleman et al., 2018). Reflectance patterns can be compared to an age matched male control (Figures 2B,D). In this subject the maximum intensity projection slab, using the same bounding as the patient, shows a uniform reflectance characteristic of having an intact ellipsoid zone over the entire region.

**FIGURE 3:**
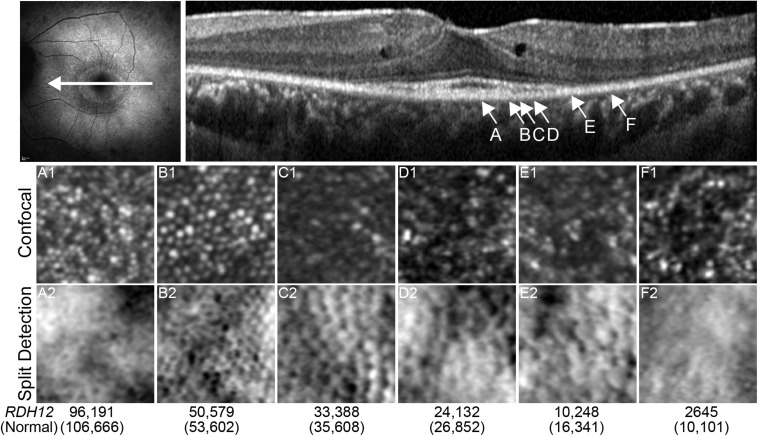
Multimodal Imaging of *RDH12* Retinopathy. Top panel shows short wavelength autofluorescence pattern, the white arrow represents the location of the SD-OCT scan. Arrows **(A–F)** are locations where AOSLO images were acquired and cone density measured. These locations are shown below. Confocal imaging reveals the intact, waveguiding outer segments while the split detection resolves residual inner segments of cone photoreceptors. Cone density between the *RDH12* patient and (normal) is shown in the bottom, with slightly reduced density at the fovea and drastically reduced density moving away from the fovea.

There was salient hypo- and hyper-autofluorescence rings present on short-wavelength fundus autofluorescence (FAF) and residual foveal island on SD-OCT (Figure 3). Parafoveal macular edema was present in both eyes. Adaptive optics scanning laser ophthalmoscopy (AOSLO) using a bespoke system with multi-detector schemes was performed on both eyes (Dubra et al., 2011; Scoles et al., 2014). Foveal cone morphology and density was relatively normal (Figure 3). Cone density remained relatively preserved across the residual OCT ellipsoid zone (EZ) island, confirmed by cellular structures present in the split detection images. However, confocal imaging showed a drastic reduction in normal appearing foveal cones (bright Gaussian profile spots), even in areas without visible macular edema (Figure 3). Split detection imaging also suggested some cellular structures outside the EZ island. Intact structures can be inferred from the round, dimple-like structures present in the split detection view.

The patient underwent whole exome sequencing and was found to have a heterozygous deletion in *RDH12* (NM_152443.2), c.759del; p.(Phe254Leufs^∗^24), which segregated with his affected father and sister, and was confirmed by Sanger sequencing (Figure 1B). This variant has not been reported on gnomAD. The pathogenicity of the variant was scored with SIFT_Indel (Hu and Ng, 2013), Variant Effect Predictor (McLaren et al., 2016) and MutationTaster (Schwarz et al., 2014), and all classified the variant as damaging or disease causing. No disease causing mutations were found in any other known retinal disease genes.

**FIGURE 4:**
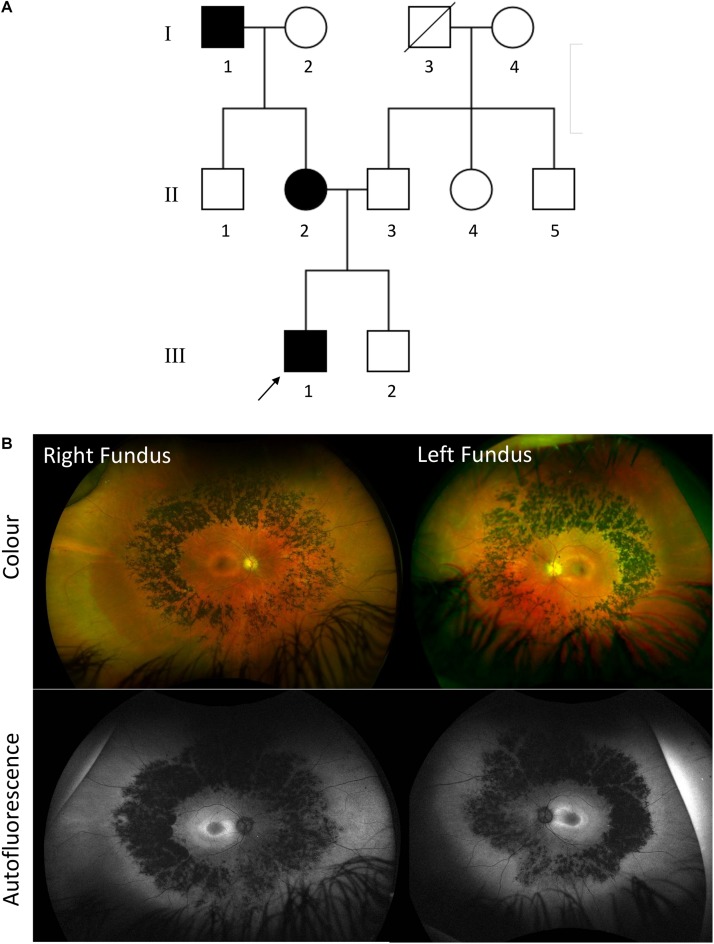
**(A)** Family pedigree of patient 2 affected with autosomal dominant *RDH12*-retinitis pigmentosa (RP). Affected individuals are colored in black. Deceased individuals are indicated with a slash and patient 2 is indicated by an arrow. **(B)** Ultrawidefield Optos color images of right and left fundus of patient 2 with corresponding autofluorescence.

Patient 2 is an unrelated 37-year-old Caucasian English man with a maternal family history of RP with an affected mother and maternal grandfather. He had a history of keratoconus from adolescence, and at age 24 has an episode of left hydrops which required a penetrating keratoplasty. After this he began to notice nyctalopia and progressive visual field loss. He maintains good central and color vision. His best corrected visual acuity is 0.18 LogMAR in both eyes and 17/17 color vision using Ishihara. Fundus examination showed mild waxy disc pallor with retinal vessel attenuation and mid-peripheral bone-spicules similar to patient 1 (Figure 4). Ultra-widefield retinal autofluorescence imaging displayed the characteristic hypo- and hyper-autofluorescence rings around the residual foveal island. Whole exome sequencing revealed the same heterozygous variant as patient 1, which segregated with his affected mother.

The mutation is located toward the end of the reading frame, with 80% of the sequence unchanged, including the two highly conserved catalytic domains: the cofactor binding site at positions 46–52, and the active site at 200–204, possibly accounting for the relatively mild phenotype (Figure 1C). This mutation results in loss of the terminal 63 amino acids, which are highly conserved across species, causing premature termination at codon 277. Due to the proximity of the mutation to the final exon–exon junction, this transcript is likely to escape nonsense mediated decay (NMD) and result in the expression of a truncated protein. This variant is only 17 bp upstream from the previously reported heterozygous variant, and both give rise to a 277 amino acid protein, with a common 17 amino acid C-terminal sequence (Figure 1C).

## Discussion

Here, we describe two unrelated families with a novel autosomal dominant variant in *RDH12*, displaying a RP phenotype (Gill et al., 2019). In patients with autosomal recessive *RDH12* variants, Aleman et al. (2018) described a rapidly progressing condition leaving only residual islands intact by the second decade of life with very poor visual acuity. The cases presented here are in stark contrast to this classical representation with relatively intact macular structure and visual acuity. The comparison is further made by the presence of the classic RP autofluorescent ring pattern compared to the much more variegated pattern in previous *RDH12* reports (Kumaran et al., 2017; Aleman et al., 2018).

Eighty *RDH12* mutations have been reported to date, 64% of which are missense, 15% are nonsense and 14% are small insertions or deletions, including 6 autosomal recessive deletions associated with the severe early onset LCA phenotype (HGMD public database accessed August 2019). A 5bp deletion in the terminal exon, c.806_810del; Ala269Glyfs^∗^2, has been reported in patients with autosomal recessive LCA, which was shown to result in loss of function, with less than 5% enzyme activity seen *in vitro* (Sun et al., 2007). This variant results in a truncated protein with only two out-of-frame amino acids, as opposed to the identical 17 out-of-frame amino acids seen in both dominant variants. This C-terminal peptide sequence produced by the dominant variants may be responsible for a protein interaction resulting in gain of function or a dominant negative effect, which may account for the milder phenotype. However, further functional studies into the differences in disease mechanisms between autosomal recessive and autosomal dominant mutations are required.

## Data Availability Statement

The datasets generated for this study can be found in ClinVar (SCV001164601).

## Ethics Statement

Written informed consent was obtained from the individuals for the publication of any potentially identifiable images or data included in this article.

## Author Contributions

HS performed the experiments for Sanger sequencing validation of mutation. AD performed ophthalmic imaging of the patients. MM and SD conducted the clinical evaluation of the patients. HS, AD, and MM wrote the manuscript.

## Conflict of Interest

The authors declare that the research was conducted in the absence of any commercial or financial relationships that could be construed as a potential conflict of interest.
